# A distal anterior cerebral artery aneurysm arising from the middle of three A2 branches treated by stent-assisted coiling: a case report

**DOI:** 10.3389/fsurg.2026.1888939

**Published:** 2026-08-11

**Authors:** Hexi Guo, Nai Zhang, Yuchang Wang, Bangyue Wang, Yang Li, Xinyu Yang

**Affiliations:** 1 Department of Neurosurgery, Tianjin Medical University General Hospital, Tianjin Medical University, Tianjin, China; 2 Department of Neurosurgery, Ordos Central Hospital, Kangbashi, China; 3 Department of Neurosurgery, The Second Affiliated Hospital of Anhui Medical University, Hefei, China

**Keywords:** A2 segment, anterior cerebral artery variation, distal anterior cerebral artery aneurysm, endovascular treatment, stent-assisted coiling

## Abstract

Distal anterior cerebral artery aneurysms (DACA) associated with complex anatomical variations of the anterior cerebral artery (ACA) are exceptionally rare. We report a rare case of a 78-year-old woman with a rare ACA configuration characterized by three distinct A2 branches, in which a saccular aneurysm developed at the bifurcation of the middle branch. The patient was admitted with sudden onset of headache, dizziness, nausea, vomiting, and hypertension. Computed tomography (CT) revealed subarachnoid hemorrhage. Computed tomography angiography (CTA) and digital subtraction angiography (DSA) revealed three distinct A2 branches arising from the ACA complex, with an aneurysm originating from the middle branch. On the eighth day after hemorrhage, stent-assisted coil embolization was successfully performed. Considering the close anatomical relationship between the aneurysm neck and the right superior trunk, a stent was strategically deployed in the right superior trunk to preserve distal cerebral perfusion. The postoperative course was uneventful, and clinical follow-up demonstrated an excellent neurological outcome. This case highlights the critical role of precise preoperative imaging evaluation (CTA, DSA) and individualized treatment strategies in managing complex vascular variations and associated aneurysms.

## Introduction

The anterior cerebral artery (ACA) is an important component of the anterior circulation of the Circle of Willis, and its anatomical variations are not uncommon in clinical practice. Anatomical variations of the ACA are common in clinical practice and represent a broad spectrum of developmental configurations. A large systematic review involving 85,316 patients demonstrated considerable morphological diversity of the ACA and anterior communicating artery (AComA) complex (1). In a magnetic resonance angiography study of 581 adults, the typical ACA–AComA configuration was observed in only 40.6% of individuals, whereas AComA aplasia (18.9%) and hypoplasia (17.9%) were among the most common variants (2). In addition, among 60 cadaveric brain specimens, the incidence of the median anterior cerebral artery (median ACA) was 11.7%, and that of the bihemispheric anterior cerebral artery (bihemispheric ACA) was 20.0% (3). Configurations involving three A2 branches have also been reported, although they are considerably less common than the typical bilateral A2 configuration (4).

Distal anterior cerebral artery aneurysms (DACA aneurysms), defined as aneurysms arising from the A2–A5 segments (5), account for approximately 1%–9% of all intracranial aneurysms (6). Although uncommon, these aneurysms are frequently associated with anatomical variations of the ACA, suggesting that vascular geometry may play an important role in their development. Geometrical alterations of cerebral arteries can substantially influence local blood flow patterns and wall shear stress, thereby contributing to aneurysm initiation and progression (7). Previous studies have demonstrated that A1 asymmetry and a smaller A1–A2 angle are closely associated with aneurysm formation in the anterior communicating artery region (8). Furthermore, variations of the Circle of Willis have been associated with an increased risk of intracranial aneurysm formation and rupture (9). These findings suggest that alterations in vascular configuration may redistribute cerebral blood flow, resulting in chronic hemodynamic stress on vulnerable arterial segments.

From an embryological perspective, the development of the ACA and AComA is tightly correlated, and the fusion and differentiation processes of embryonic cerebral vessels determine the spectrum of anatomical variants observed in adulthood (10). Among these variations, the classic description of ACA triplication consists of three A2 segments arising from a well-developed AComA, with the third vessel commonly referred to as the accessory anterior cerebral artery or median callosal artery (11). This configuration has been reported as one of the rare distal ACA variants, together with the azygos ACA and bihemispheric ACA (12). Rare vascular configurations involving three A2 branches have also been reported in association with intracranial aneurysms, presumably because altered vascular geometry modifies local hemodynamic conditions (13).

The presence of uncommon distal ACA branching patterns presents additional challenges for endovascular treatment. The complex anatomy of the AComA complex, the relationship between the bilateral A1 and A2 segments, the presence of perforating arteries or AComA fenestration, and the spatial relationship between the aneurysm and adjacent branches all require meticulous preoperative evaluation (14). Careful assessment of these anatomical features facilitates appropriate selection of treatment strategy while minimizing the risk of branch occlusion and ischemic complications. ACA variations may therefore substantially increase the technical complexity of endovascular management for DACA aneurysms (15).

We report a rare case of a distal ACA aneurysm arising from the middle of three distinct A2 branches, successfully treated with stent-assisted coil embolization. This case highlights the value of detailed preoperative imaging assessment and individualized endovascular planning in the management of distal ACA aneurysms associated with rare ACA branching configurations.

## Case report

**Figure 1:**
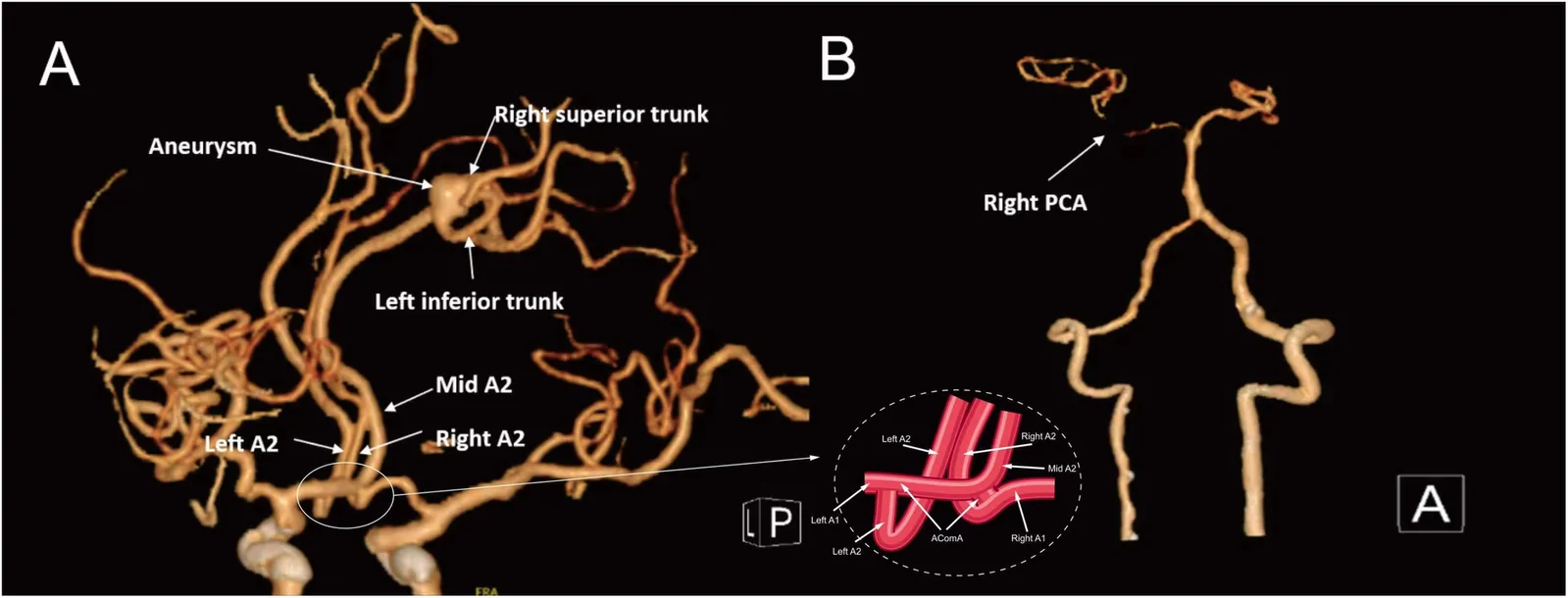
**(A)** Left posterolateral view showing a triplicated anterior cerebral artery with an aneurysm at the distal portion of the middle branch, the distal end of the middle A2 branch gives rise to the right superior trunk and the left inferior trunk. The white circled region highlights the triplicated A2 complex, with an explanatory schematic magnified in the lower right inset to delineate the anatomy of bilateral A1, triplicated A2 segments (Left A2, Mid A2, Right A2) and anterior communicating artery (AComA). **(B)** Frontal view demonstrates poor visualization of the right posterior cerebral artery.

A 78-year-old female was admitted to our hospital due to headache, dizziness, nausea and vomiting, along with elevated blood pressure, which occurred 3 days ago without an obvious cause. Physical examination: no obvious positive findings. Radiological examination: Computed tomography (CT) revealed subarachnoid hemorrhage. Computed tomography angiography (CTA) demonstrated three distinct A2 branches, with a saccular aneurysm located at the distal portion of the middle branch. The distal end of the middle A2 branch gives rise to the right superior trunk and the left inferior trunk (Figure 1A). The right posterior cerebral artery shows poor visualization (Figure 1B).

**Figure 2:**
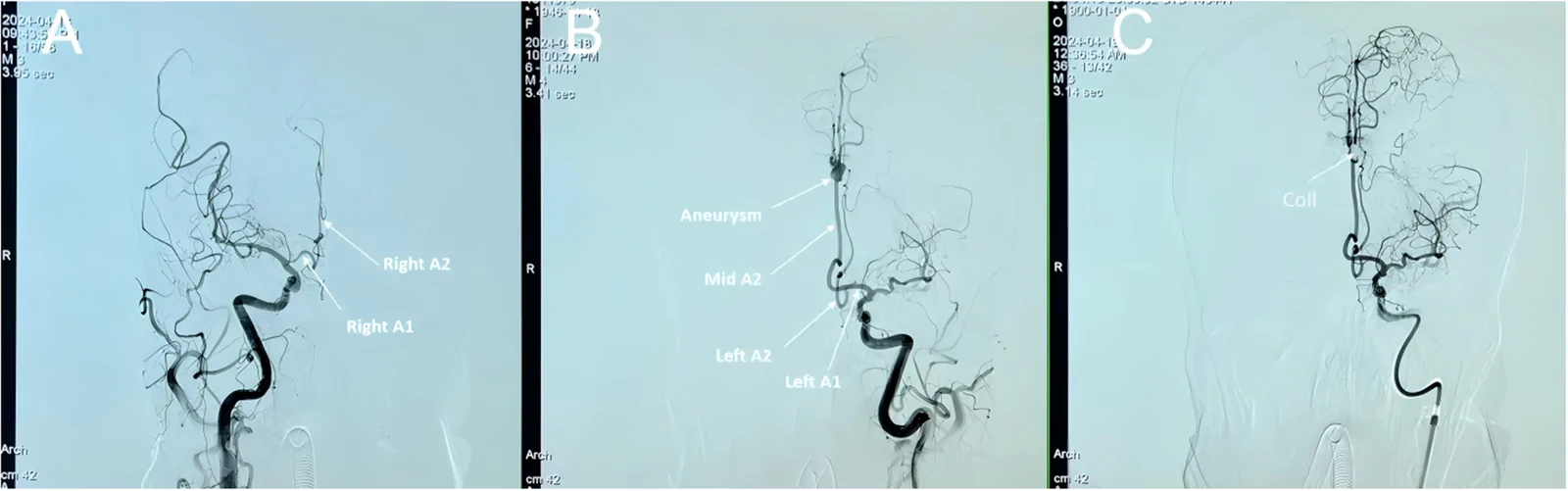
**(A)** Right internal carotid artery angiography revealed that the right A1 segment of the anterior cerebral artery (ACA) supplied only the right A2 branch. **(B)** Left internal carotid artery angiography showed that the left A1 segment supplied both the middle and left A2 branches. The aneurysm was located at the distal bifurcation of the middle A2 branch. The distal A2 gave rise to the right superior trunk and the left inferior trunk, which further bifurcated to supply both cerebral hemispheres. **(C)** The aneurysm was successfully coiled, and the normal vessels remained unaffected.

**Figure 3:**
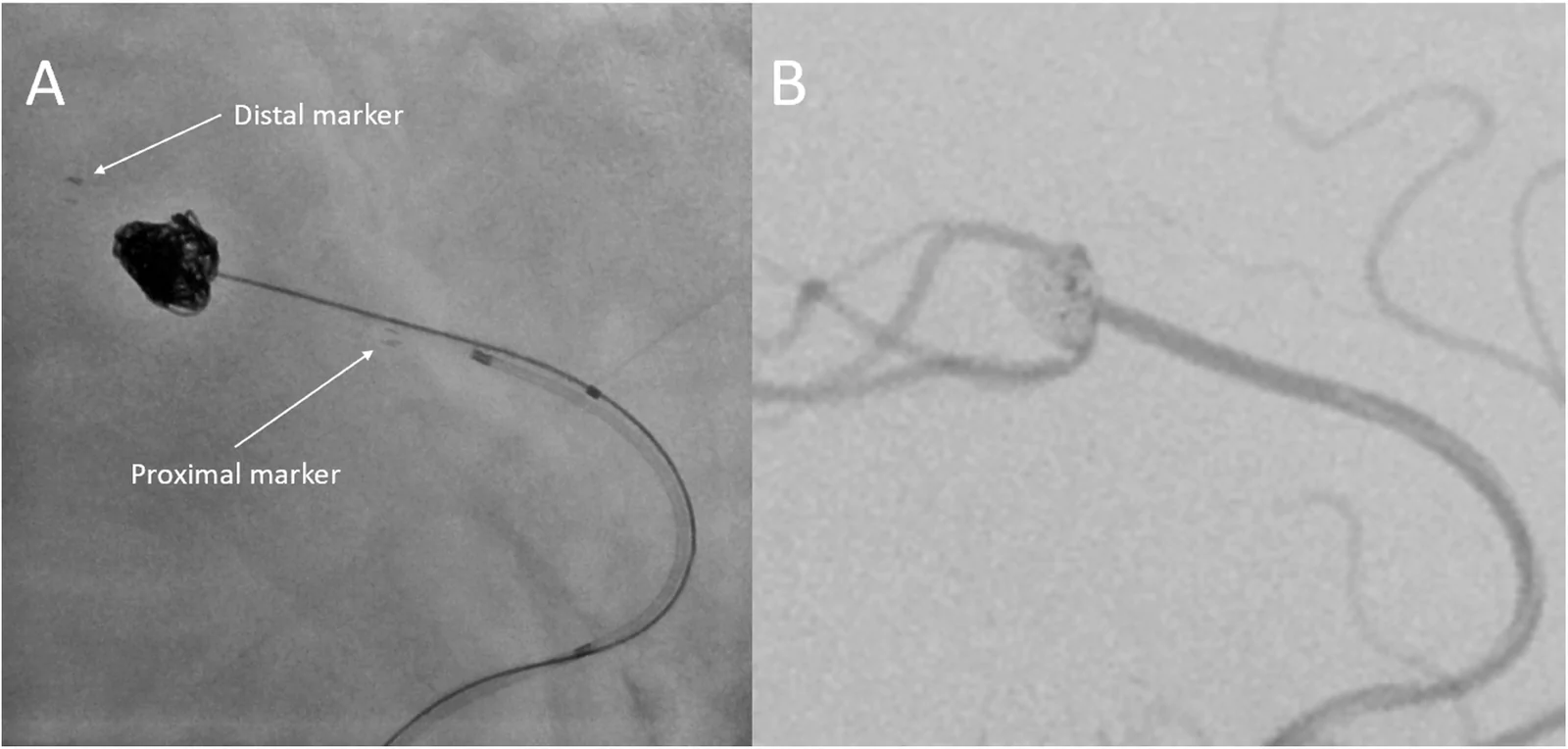
**(A)** Magnified native fluoroscopic image obtained during the final stage of coil deployment, demonstrating the coil mass and the proximal and distal stent markers. **(B)** Final subtracted DSA image demonstrating complete aneurysm occlusion with preservation of the parent artery and distal branches.

**Figure 4:**
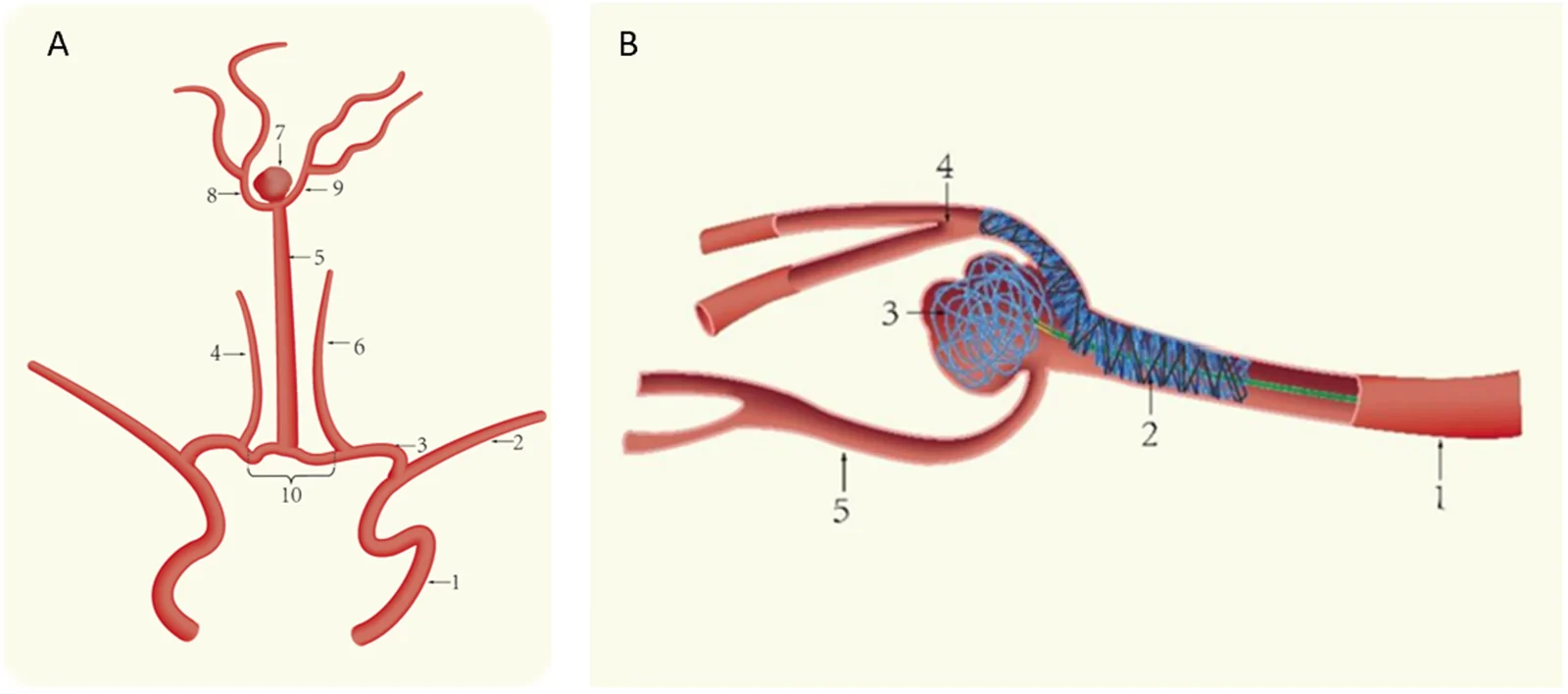
**(A)** Schematic design illustrating artery anatomy variation of a triplication of the anterior cerebral artery. (1) Left ICA, (2) left MCA, (3) left A1, (4) right A2, (5) mid A2, (6) left A2, (7) aneurysm, (8) right superior trunk, (9) left inferior trunk, (10) AComA. **(B)** Schematic design illustrating stent-assisted coil embolization. (1) Mid A2, (2) stent, (3) coil, (4) right superior trunk, (5) left inferior trunk.

Operation: Endovascular coiling of the intracranial aneurysm was performed on day 8 after hemorrhage. Intraoperative angiography revealed that the right A1 segment of the ACA supplied only the right A2 branch (Figure 2A), whereas the left A1 segment supplied both the middle and left A2 branches (Figure 2B). The aneurysm was located at the distal bifurcation of the middle A2 branch. The distal A2 gave rise to the right superior trunk and the left inferior trunk, which further bifurcated to supply both cerebral hemispheres (Figures 2B, 4A). Representative magnified fluoroscopic and subtracted angiographic images obtained during and after embolization are shown in Figure 3, demonstrating the coil configuration, stent position, and complete aneurysm occlusion. During the procedure, stent-assisted coil embolization was performed. Considering the close anatomical relationship between the aneurysm neck and the right superior trunk, a laser-cut Enterprise stent (Codman Neuro, USA) was strategically deployed in the superior trunk to preserve medial cerebral perfusion (Figure 4B).

The rationale for placing the stent in the superior trunk was as follows:

First, the aneurysm neck was closely related to the superior trunk, and visualization of the right posterior cerebral artery was poor. Considering that occlusion of the right superior trunk could result in severe ischemia of the right medial cerebral surface, stent protection of this branch was prioritized. Second, the left inferior trunk was less involved by the aneurysm and showed good opacification. Therefore, it had a higher likelihood of remaining patent without stent protection. Additionally, collateral flow from the posterior circulation through the left posterior cerebral artery could provide compensatory perfusion to the left medial surface. Even if the inferior trunk became occluded intraoperatively, significant infarction of the left medial cerebral surface would be unlikely. The aneurysm was successfully coiled (Figures 2C, 3). The post operative course was uneventful, with complete recovery. Postoperative CT scan was normal. Clinical follow-up was performed on June 14, 2024, and April 25, 2025. At the latest follow-up, the patient remained neurologically intact with an mRS score of 0 and no recurrent neurological symptoms. Follow-up vascular imaging was performed at another institution and was unavailable for review.

## Discussion

This case reports a rare distal anterior cerebral artery aneurysm arising from the middle of three distinct A2 branches, successfully treated by stent-assisted coil embolization, highlighting its embryologic origin, clinical relevance, and treatment considerations.

Rare distal ACA branching configurations involving three A2 branches have been reported in the literature and represent uncommon vascular developmental variations. Several studies have documented the occurrence and associated features of this variation, highlighting its rarity and potential clinical relevance. A study reported an exceptionally rare anatomical variation characterized by both triplication of the ACA and duplication of the AComA, underscoring the complexity and rarity of such vascular configurations (16). Similarly, Previous studies have described cases of fetal posterior cerebral artery (fPCA) duplication occurring alongside ACA triplication, highlighting the developmental variability of cerebral arterial anatomy (17).

Furthermore, previous anatomical studies have suggested that uncommon distal ACA branching patterns, including those described as ACA triplication, may be associated with aneurysm formation. A study analyzed the features of distal ACA aneurysms and noted that anomalies such as ACA variations, including triplication, are associated with small-sized aneurysms with broad bases and originating branches (18). Such variations can complicate surgical and endovascular interventions.

Other reports have documented uncommon ACA and cerebrovascular anatomical variations and their clinical relevance. A study reported a ruptured distal accessory anterior cerebral artery aneurysm arising from a rare ACA anatomical variant, highlighting the association between distal ACA aneurysms and uncommon ACA branching configurations (19). Another anatomical study investigated variations of the Circle of Willis and the anterior communicating artery, demonstrating that anatomical variations of the ACA and AComA are not uncommon and should be recognized during the evaluation of cerebrovascular anatomy (20). Similarly, a separate case report described triplication of the middle cerebral artery associated with fenestration of the anterior cerebral artery, further illustrating the coexistence of multiple congenital cerebrovascular anomalies (21).

The anatomical classification of the present case deserves careful consideration. Although the vascular configuration observed in this patient shares several features with the classic description of ACA triplication reported in the literature (11), the origin of the third A2 branch differs from the classic anatomical pattern, and therefore its anatomical classification warrants careful consideration. Regardless of the terminology adopted, the present case demonstrates a rare distal ACA branching configuration with three distinct A2 branches that has important implications for aneurysm formation and endovascular treatment planning.

Although anatomical and clinical studies have reported an association between uncommon distal ACA branching configurations—including those described as ACA triplication—and distal aneurysms (18, 19), the underlying hemodynamic mechanisms remain incompletely understood. Abnormal hemodynamic stress is widely considered one of the principal mechanisms underlying intracranial aneurysm formation (22). Although direct hemodynamic evidence for the present vascular configuration is lacking, altered vascular geometry associated with rare ACA branching configurations may also influence local blood-flow patterns and wall shear stress. The potential hemodynamic implications of this vascular variant are discussed below.

Abnormally altered hemodynamic wall shear stress (WSS) at arterial bifurcations is widely considered one of the principal mechanisms underlying intracranial aneurysm initiation (22). WSS is primarily determined by local vascular geometry, including bifurcation configuration, vessel diameter, and branching angles (7). Because alterations in vascular geometry substantially influence local flow patterns, anatomical variants may modify the hemodynamic environment throughout the ACA circulation. In vascular configurations characterized by three A2 branches, three distal ACA trunks may arise from the anterior communicating artery complex or adjacent ACA segments, creating additional flow-dividing junctions and altering the geometric configuration of the proximal ACA. These structural changes may redistribute blood flow within the ACA system and generate heterogeneous WSS and wall shear stress gradient (WSSG) patterns, particularly when branch calibers and branching angles are asymmetric (23). Previous studies have shown that wider bifurcation angles and smaller branch diameters are associated with increased maximal WSS and a larger area exposed to high WSS (24). Such hemodynamic alterations may promote endothelial dysfunction and vascular remodeling (23), thereby increasing susceptibility to aneurysm formation at vulnerable arterial bifurcations rather than exclusively at the site of the anatomical variant. Although computational fluid dynamics analysis was not performed in the present case, these mechanisms provide a plausible explanation for aneurysm development in this rare vascular variant.

Although the aneurysm in the present case arose at a distal ACA bifurcation rather than within the anterior communicating artery complex, it is conceivable that proximal ACA branching variations could alter blood-flow distribution within the ACA circulation. Computational fluid dynamics studies have demonstrated that alterations in ACA vascular geometry, particularly asymmetric A1 segments and changes in the A1–A2 configuration, can modify local blood-flow patterns and wall shear stress within the ACA system (25). Because vascular geometry is a major determinant of local hemodynamics, the presence of three distinct A2 branches in the present case may similarly influence flow distribution and wall shear stress, although this hypothesis has not yet been validated by patient-specific computational fluid dynamics analysis. Accordingly, the hemodynamic consequences of proximal anatomical variations may not be restricted to the immediate site of the variation but could potentially extend to downstream bifurcations, thereby contributing to aneurysm formation at susceptible distal locations. However, because patient-specific computational fluid dynamics analysis was not performed, this proposed mechanism remains speculative and warrants further investigation.

Endovascular stent-assisted coil embolization was performed after detailed vascular evaluation. Intraoperative angiography showed the aneurysm neck was adjacent to the right superior trunk; a protective stent was placed to prevent ischemia, while the well-perfused left inferior trunk required no stent. This individualized strategy highlights the importance of meticulous preoperative assessment of vascular anatomy, branch relationships, and aneurysm morphology in planning endovascular treatment (14, 15, 18). This case demonstrates that precise understanding of vascular variations—supported by modern imaging such as DSA and CTA—is crucial for safe, effective management (15). Because vascular geometry is a major determinant of local hemodynamic conditions, rare ACA branching configurations involving three A2 branches may likewise modify local blood-flow patterns and wall shear stress, thereby potentially contributing to aneurysm formation (7, 22, 23). Successful treatment underscores the value of comprehensive imaging and tailored endovascular planning to achieve complete occlusion while preserving cerebral perfusion (15). Although clinical follow-up demonstrated an excellent neurological outcome, the lack of available follow-up angiographic images limits assessment of the long-term durability of aneurysm occlusion.

## Conclusion

Rare distal anterior cerebral artery branching configurations involving three distinct A2 branches may be associated with aneurysm formation and presents significant challenges for endovascular management. This case demonstrates that detailed preoperative evaluation with CTA and DSA is essential for accurately defining complex vascular anatomy and collateral circulation. Individualized stent-assisted coil embolization enabled complete aneurysm occlusion while preserving critical cerebral perfusion. Careful assessment of branch anatomy, aneurysm morphology, and local hemodynamic characteristics is crucial for selecting an optimal treatment strategy in patients with rare cerebrovascular anomalies.

## Data Availability

The original contributions presented in the study are included in the article/Supplementary Material, further inquiries can be directed to the corresponding author.
